# Comparative Evaluation of a Novel Recombinase Polymerase Amplification-Lateral Flow Dipstick (RPA-LFD) Assay, LAMP, Conventional PCR, and Leaf-Disc Baiting Methods for Detection of *Phytophthora sojae*

**DOI:** 10.3389/fmicb.2019.01884

**Published:** 2019-08-09

**Authors:** Tingting Dai, Xiao Yang, Tao Hu, Binbin Jiao, Yue Xu, Xiaobo Zheng, Danyu Shen

**Affiliations:** ^1^College of Forestry, Co-Innovation Center for the Sustainable Forestry in Southern China, Nanjing Forestry University, Nanjing, China; ^2^Foreign Disease-Weed Science Research Unit, USDA, Agricultural Research Service, Fort Detrick, MD, United States; ^3^ARS Research Participation Program, Oak Ridge Institute for Science and Education, Oak Ridge, TN, United States; ^4^Technical Center for Animal, Plant and Food Inspection and Quarantine of Shanghai Customs, Shanghai, China; ^5^Department of Plant Pathology, Nanjing Agricultural University, Nanjing, China

**Keywords:** oomycetes, plant destroyers, field diagnosis, *Phytophthora sansomeana*, *Phytophthora melonis*, *Phytophthora vignae*

## Abstract

Early and accurate detection of the causal pathogen *Phytophthora sojae* is crucial for effective prevention and control of root and stem rot and seedling damping-off of soybean. In the present study, a novel isothermal amplification assay was developed for detecting *P. sojae*. This 25 min assay included a two-step approach. First, a pair of novel primers, PSYPT-F and PSYPT-R were used to amplify a specific fragment of the *Ypt1* gene of *P. sojae* in a 20 min recombinase polymerase amplification (RPA) step. Second, lateral flow dipsticks (LFD) were used to detect and visualize RPA amplicons of *P. sojae* within 5 min. This RPA-LFD assay was specific to *P. sojae*. It yielded negative detection results against 24 other *Phytophthora*, one *Globisporangium*, and 14 fungal species. It was also found to be sensitive, detecting as low as 10 pg of *P. sojae* genomic DNA in a 50-μL reaction. Furthermore, *P. sojae* was detected from artificially inoculated hypocotyls of soybean seedlings using this novel assay. In a comparative evaluation using 130 soybean rhizosphere samples, this novel assay consistently detected *P. sojae* in 55.4% of samples, higher than other three methods, including loop-mediated isothermal amplification (54.6%), conventional PCR (46.9%), and leaf-disc baiting (38.5–40.0%). Results in this study indicated that this rapid, specific, and sensitive RPA-LFD assay has potentially significant applications to diagnosing Phytophthora root and stem rot and damp-off of soybean, especially under time- and resource-limited conditions.

## Introduction

*Phytophthora sojae* is one of the most devastating pathogens of soybean crops (*Glycine max*), causing damping-off on seedlings and root and stem rot on older plants. Areas that receive heavy rain may suffer plant mortality and yield losses up to 100% (Tyler, 2007; Dorrance, 2018). An estimated annual worldwide loss of 1–2 billion U.S. dollar has been caused by this pathogen (Wrather and Koenning, 2006; Tyler, 2007). *P. sojae* was first reported as a novel causal pathogen of soybean root and stem rot in Indiana and Ohio, United States (Kaufmann and Gerdemann, 1958). Thereafter, it has become widespread in many soybean-producing countries (Schmitthenner, 1985; Erwin and Ribeiro, 1996). After assessing its potential risks to agricultural and economic security, the Ministry of Agriculture of the People’s Republic of China identified *P. sojae* as a quarantine pest in 2007^1^, whereas it was discovered in Jilin and Heilongjiang Provinces in 1989 (Su and Shen, 1993). Spread of this pathogen has been accelerated by China’s increasing international and interprovincial trade and transportation of soybean seeds and plants (Cui et al., 2010; Wu et al., 2017). To date, the pathogen has been found in the Inner Mongolia Autonomous Region, Xinjiang Uygur Autonomous Region, Huanghe-Huaihe River Basin and Yangtze River Basin (Chen and Wang, 2017), as well as Jilin, Heilongjiang (Su and Shen, 1993), Fujian (Cui et al., 2010; Wu et al., 2017), and Anhui (Dai Y.L. et al., 2015) Provinces.

Rapid detection of *P. sojae* is a crucial step toward effective management of soybean root and stem rot and seedling damping-off. Traditionally, detection methods for *P. sojae* include isolation from symptomatic plant tissues and baiting from soil (Erwin and Ribeiro, 1996). Subsequent pathogen identification based on morphological characters and DNA sequence data is usually time-consuming and requires trained expertise. A variety of molecular detection methods including conventional PCR (Wang et al., 2006; Bienapfl et al., 2011; Xiong et al., 2019), quantitative PCR (Wang et al., 2006; Bienapfl et al., 2011; Haudenshield et al., 2017), LAMP assays (Dai et al., 2012, Dai Y.L. et al., 2015), and a recombinase polymerase amplification (RPA) assay targeting the *atp9*–*nad9* region of the mitochondrial genome (Rojas et al., 2017) have been developed for *P. sojae*. However, field application of PCR-based methods is limited due to their long time span and requirement for thermocyclers and gel electrophoresis. Furthermore, specificity to *P. sojae* of previously developed methods has been challenged by newly emerging pathogens (Rojas et al., 2017; Xiong et al., 2019), such as *P. sansomeana* (Hansen et al., 2009), also a pathogen of soybean, and *P. melonis* and *P. vignae*, two sister species phylogenetically related to *P. sojae* (Yang et al., 2017). Thus, a rapid and *P. sojae*-specific method that can be performed under time- and resource-limited conditions is warranted.

In the present study, a novel RPA assay targeting the *Ypt1* gene of *P. sojae* was developed. The RPA amplicons were designed to be detected using lateral flow dipsticks (LFD) in real-time. Additionally, specificity to *P. sojae* of this assay was validated by testing against *P. sansomeana*, *P. melonis*, *P. vignae*, and other oomycete and fungal species.

## Materials and Methods

### Isolate Selection of *Phytophthora* Species

Twenty-nine isolates of *P. sojae* were tested in this study (Table 1). The 11 isolates with determined pathotypes (races) including R2, R3, R6, R8, R12, R14, R17, R19, R20, R28, and R31 were provided by Dr. Brett Tyler at Oregon State University, United States and Dr. Jinhuo Peng at Dalian Animal and Plant Quarantine Bureau, China (Table 1). The remaining 18 *P. sojae* isolates were recovered from root and stem tissues of diseased soybean crops in Jiangsu, Fujian, and Yunnan Provinces, China. Isolates belonging to 24 other *Phytophthora*, one *Globisporangium*, and 14 fungal species were used for specificity evaluation. All isolates were maintained in collections at Department of Plant Pathology at Nanjing Agricultural University and Department of Forest Protection at Nanjing Forestry University in Nanjing, China.

**TABLE 1:** List of oomycete and fungal isolates used in this study and their detection results in the recombinase polymerase amplification-lateral flow dipstick (RPA-LFD) assay.

**Species (Pathotype/Race)**	**Isolate**	**Origin^a^**		**RPA-LFD^c^**
		**Host/substrate**	**Location^b^**	
*Phytophthora sojae* (R2)	P6497	*Glycine max*	Mississippi, United States	+
*P. sojae* (R3)	Peng-R3	*Glycine max*	n.a.	+
*P. sojae* (R6)	Peng-R6	*Glycine max*	n.a.	+
*P. sojae* (R8)	Peng-R8	*Glycine max*	n.a.	+
*P. sojae* (R12)	Peng-R12	*Glycine max*	n.a.	+
*P. sojae* (R14)	Peng-R14	*Glycine max*	n.a.	+
*P. sojae* (R17)	P7074	*Glycine max*	Mississippi, United States	+
*P. sojae* (R19)	P7076	*Glycine max*	Mississippi, United States	+
*P. sojae* (R20)	Peng-R20	*Glycine max*	n.a.	+
*P. sojae* (R28)	Peng-R28	*Glycine max*	n.a.	+
*P. sojae* (R31)	Peng-R31	*Glycine max*	n.a.	+
*P. sojae*	Ps1	*Glycine max*	JS, China	+
*P. sojae*	Ps2	*Glycine max*	JS, China	+
*P. sojae*	Ps3	*Glycine max*	JS, China	+
*P. sojae*	Ps4	*Glycine max*	JS, China	+
*P. sojae*	Ps5	*Glycine max*	JS, China	+
*P. sojae*	Psf1	*Glycine max*	FJ, China	+
*P. sojae*	Psf2	*Glycine max*	FJ, China	+
*P. sojae*	Psf3	*Glycine max*	FJ, China	+
*P. sojae*	Psf4	*Glycine max*	FJ, China	+
*P. sojae*	Psf5	*Glycine max*	FJ, China	+
*P. sojae*	Psy1	*Glycine max*	YN, China	+
*P. sojae*	Psy2	*Glycine max*	YN, China	+
*P. sojae*	Psy3	*Glycine max*	YN, China	+
*P. sojae*	Psy4	*Glycine max*	YN, China	+
*P. sojae*	Psy5	*Glycine max*	YN, China	+
*P. sojae*	Psy6	*Glycine max*	YN, China	+
*P. sojae*	Psy7	*Glycine max*	YN, China	+
*P. sojae*	Psy8	*Glycine max*	YN, China	+
*P. melonis*	Pme1	*Cucumis sativus*	JS, China	−
*P. vignae*	P3019	*Vigna* sp.	Australia	−
*P. sansomeana*	Yili71	*Glycine max*	XJ, China	−
*P. boehmeriae*	Pbo1	*Gossypium* sp.	JS, China	−
*P. cactorum*	Pcac1	*Malus pumila*	JS, China	−
*P. cambivora*	CBS 248.60	*Castanea sativa*	France	−
*P. capsici*	Pcap1	*Capsicum annuum*	JS, China	−
*P. cinnamomi*	Pcin1	*Cedrus deodara*	JS, China	−
*P. citrophthora*	Pcit1	*Citrus reticulata*	JS, China	−
*P. cryptogea*	Pcr1	*Gerbera jamesonii*	JS, China	−
*P. drechsleri*	CBS 292.35	*Beta vulgaris* var. *altissima*	California, United States	−
*P. erythroseptica*	CBS 129.23	*Solanum tuberosum*	Ireland	−
*P. hibernalis*	CBS 270.31	*Citrus sinensis*	Setúbal, Portugal	−
*P. infestans*	Pin1	*Solanum tuberosum*	FJ, China	−
*P. lateralis*	CBS 168.42	*Chamaecyparis lawsoniana*	Oregon, United States	−
*P. medicaginis*	ATCC 44390	*Medicago sativa*	California, United States	−
*P. megasperma*	CBS 305.36	*Matthiola incana*	California, United States	−
*P. nicotianae*	Pni1	*Nicotiana tabacum*	YN, China	−
*P. palmivora*	Ppa1	*Iridaceae* sp.	YN, China	−
*P. quercina*	CBS 789.95	Rhizosphere of *Quercus cerris*	Germany	−
*P. ramorum*	EU1 2275	*Laurus nobilis*	UK	−
*P. rubi*	CBS 967.95	*Rubus idaeus*	Scotland, United Kingdom	−
*P. syringae*	CBS 132.23	*Malus domestica*	UK	−
*P. tentaculata*	Pte1	*Saussurea costus*	YN, China	−
*Globisporangium ultimum*	Gu1	Irrigation water	JS, China	−
*Alternaria alternata*	Aal1	Soil	JS, China	−
*Botrytis cinerea*	Bci1	*Cucumis sativus*	JS, China	−
*Bremia lactucae*	Bla1	*Lactuca sativa*	JS, China	−
*Colletotrichum glycines*	Cgl1	*Glycine max*	JS, China	−
*Colletotrichum truncatum*	Ctr1	*Glycine max*	JS, China	−
*Colletotrichum orbiculare*	Cor1	*Citrullus lanatus*	JS, China	−
*Endothia parasitica*	Epa1	*Castanea mollissima*	JS, China	−
*Fusarium equiseti*	Feq1	*Gossypium* sp.	JS, China	−
*Fusarium oxysporium*	Fox1	*Gossypium* sp.	JS, China	−
*Fusarium solani*	Fso1	*Gossypium* sp.	JS, China	−
*Magnaporthe grisea*	Mgr1	*Oryza sativa*	JS, China	−
*Magnaporthe grisea*	Mgr2	*Oryza sativa*	YN, China	−
*Rhizoctonia solani*	Rso1	*Gossypium* sp.	JS, China	−
*Tilletia indica*	Tin1	*Triticum aestivum*	JS, China	−
*Verticilium dahliae*	Vda1	*Gossypium* sp.	JS, China	−

*^*a*^n.a. = not available. ^*b*^Abbreviations of provinces in China: JS, Jiangsu province; FJ, Fujian province; YN, Yunnan province; XJ, Xinjiang Uygur Autonomous Region. ^*c*^Positive (+) or negative (−) reaction result in the RPA-LFD assay for detecting *P. sojae*.*

### Culture Conditions and DNA Extraction

*Phytophthora* and *Globisporangium* isolates were cultured in 10% clarified V8 juice agar at 25°C in the dark. Fungal isolates were maintained in potato dextrose agar at 25°C in the dark.

For extracting genomic DNAs (gDNAs), each oomycete or fungal isolate was grown in 10% clarified V8 juice or potato dextrose broth, respectively, at 25°C for 4–5 days, harvested, and freeze dried. gDNAs were extracted using a DNAsecure Plant Kit (TIANGEN, Beijing, China) according to the manufacturer’s instructions. Total DNAs were extracted from artificially inoculated soybean tissues using an NaOH lysis method (Wang et al., 1993). Environmental DNAs (eDNAs) from rhizosphere samples were extracted using a FastDNA SPIN Kit for Soil (MP Biomedicals, Solon, Ohio, United States). Specifically, 400 mg of each soil sample was placed in a lysing matrix E 2-mL tube, followed by the addition of 978 mL of phosphate buffer and 122 mL of MT buffer (MP Biomedicals, Solon, OH, United States). Mixtures in lysing tubes were homogenized using a FastPrep FP120 instrument (MP Biomedicals, Solon, OH, United States) at speed 6 for 40 s. Extraction of eDNAs was completed following manufacturer’s instructions.

DNA concentrations were quantified using a NanoDrop 1000 spectrophotometer (Thermo Fisher Scientific, Wilmington, DE, United States). All DNA extractions were stored at −20°C until use.

### Primers and Probe Design

**FIGURE 1:**
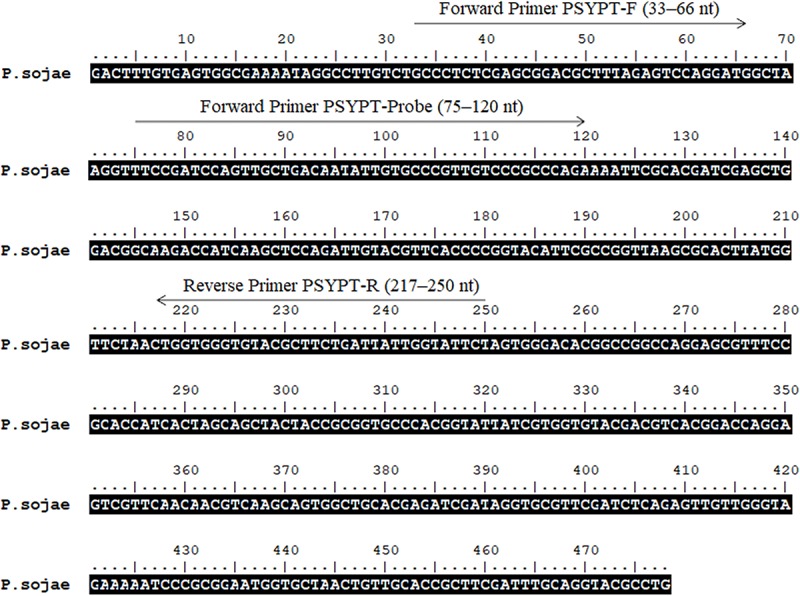
Sequence of the *Ypt1* gene of *Phytophthora sojae* (GenBank accession No. DQ162958). Nucleotides targeted by forward primers PSYPT-F, PSYPT-Probe, and reverse primer PSYPT-R of the novel recombinase polymerase amplification assay are below respective arrows. Arrows indicate the direction of amplification. Primer sequences are provided in Table 2.

Sequences of the *Ypt1* gene of *P. sojae* (GenBank accession No. DQ162958) and its phylogenetically close species were downloaded from GenBank (Benson et al., 2018). Multiple sequence alignment by Clustal W (Larkin et al., 2007) was carried out using BioEdit version 7.0.5 (Hall, 1999). Several combinations of RPA primers and probe targeting the *P. sojae*-specific fragment within the 478-nt sequence (Figure 1) were designed according to RPA guidelines and manufacturer’s instructions for Twist Amp^®^ DNA amplification kit (TwistDx Ltd., Cambridge, United Kingdom), followed by testing in RPA to identify the optimal primer set. A pair of forward primer and a 5′-biotin-labeled reverse primer (Table 2) met the requirement for the specific detection of *P. sojae Ypt1* gene according to the TwistAmp^®^ nfo kit (TwistDx Ltd., Cambridge, United Kingdom). Thereafter, a nfo DNA probe (Table 2) used for the LFD visualization (Milenia Biotec, Giessen, Germany) was designed based on the sequences of RPA primers. This nfo probe was labeled with a fluorescein amidite (FAM) at the 5′ end, a base analog tetrahydrofuran (THF) inserted between the 30th and 31st bases, and a C3 spacer at the 3′ end (Table 2). The primers and probe (Table 2) were synthesized by GenScript (Nanjing, China).

**TABLE 2:** Oligonucleotide primers and probe designed for the recombinase polymerase amplification-lateral flow dipstick assay in this study.

**Name**	**Sequence (5′–3′)**
PSYPT-F primer	GCCCTCTCGAGCGGACGCTTTAGAGTCCAGGATG
PSYPT-R primer	[Biotin]AGAATACCAATAATCAGAAGCGTACACCCACCAG
PSYPT-Probe	[FAM]TTCCGATCCAGTTGCTGACAATATTGTGCC[THF]G
	TTGTCCCGCCCAGA[C3-spacer]

### RPA-LFD Assay

Recombinase polymerase amplification-Lateral flow dipsticks assay was performed according to the quick guide of TwistAmp^®^ nfo kit (TwistDx Ltd., Cambridge, United Kingdom). Briefly, each 50 μL reaction contained 29.5 μL of rehydration buffer (supplied in the kit), 2.1 μL of each of forward and reverse primers (10 μM), 0.6 μL of probe (10 μM), 12.2 μL of nuclease-free water (nfH_2_O; Thermo Fisher Scientific, Wilmington, DE, United States), and 1 μL of DNA template. After mixing by vortex, 2.5 μL of 280-mM magnesium acetate was added to each reaction for initiating amplification. RPA was performed at 39°C in a SimpliAmp^TM^ thermal cycler instrument (Model A24812, Thermo Fisher Scientific, Wilmington, DE, United States) for 20 min with non-heated lid and a vortex and spin step after the first 4 min. To detect RPA amplicons, 10 μL of RPA product was mixed with 90 μL of phosphate buffered saline with Tween 20 (PBST) running buffer. Then 10 μL of the mixture was added to the sample pad of a HybriDetect 1 LFD (Milenia Biotec GmbH, Giessen, Germany) using a pipettor. The LFD was dipped into a tube containing 200 μL of PBST and incubated at room temperature (aver. 22°C) for up to 5 min until a control line was visible. When test and control lines were simultaneously visible, it was a positive detection. If only the control line was visible, it was a negative detection. All LFDs were then air-dried and photographed using a Canon PowerShot SX730 HS camera.

### RPA-LFD Assay Specificity and Sensitivity

Specificity of the RPA-LFD assay was evaluated against all isolates listed in Table 1. Each RPA reaction included 10 ng of purified gDNA. RPA-LFD assay was performed in triplicate against each isolate.

To determine sensitivity, 10-fold dilutions of *P. sojae* gDNA (isolate P6497) ranging from 100 to 0.001 ng per μL were used as DNA templates in the RPA-LFD assay. nfH_2_O was used in no-template control (NTC) reactions. This RPA-LFD assay was repeated in triplicate for each concentration of gDNA template under the same conditions described above.

### Detection of *P. sojae* in Artificially Inoculated Soybean Seedlings Using RPA-LFD

Seedlings of soybean cultivar Hefeng 47 were grown in a glasshouse at a day/night temperature of 25/20°C and a 16 h photoperiod. *P. sojae* isolate P6497 was cultured in rye grains mixed with 10% clarified V8 juice at 25°C in the dark for 3 days. Hypocotyls of 4-day-old soybean seedlings were wounded using a sterile inoculation needle. A *P. sojae*-colonized rye grain was placed on the wound site of each of three seedlings. A sterile grain was used for each of three non-inoculated seedlings. Hypocotyl tissues were then covered using parafilm to keep rye grains attached and maintain humidity. Development of symptoms was recorded daily. At approximately 72 h after inoculation, total gDNAs at the wounded site of hypocotyls were extracted as described above. Concentrations of gDNA extractions were measured using a NanoDrop 1000 spectrophotometer (Thermo Fisher Scientific, Wilmington, DE, United States) and adjusted to 10 ng per μL by adding nfH_2_O. The RPA-LFD assay was performed as described above using the hypocotyl total DNA extractions as templates. This experiment was repeated once. Purified gDNA (10 ng per μL) of *P. sojae* isolate P6497 and nfH_2_O were included in each repeat as a positive control and NTC, respectively.

### Comparative Evaluation of Detection Assays Using Soybean Rhizosphere Samples

One hundred and thirty rhizosphere samples (0- to 10-cm depth) were collected from soybean fields in seven cities of the Heilongjiang Province in China, namely Daqing, Haerbin, Jiamusi, Jixi, Mudanjiang, Qiqihaer, and Yichun, from 2008 to 2014 (Table 3). After sampling, they were stored in 1-gallon Ziploc bags and transported in ice boxes to laboratories at Nanjing Agricultural University and Nanjing Forestry University. eDNAs were extracted from all samples and quantified as described above.

**TABLE 3:** Detection of *Phytophthora sojae* using a novel recombinase polymerase amplification-lateral flow dipstick (RPA-LFD) assay developed in this study, and three previously used methods including Loop-mediated isothermal amplification (LAMP), conventional PCR, and leaf-disc baiting on 130 rhizosphere samples collected from soybean fields in Heilongjiang Province, China.

**Location**	**Sample size**	**No. of positives**			
		**RPA-LFD**	**LAMP**	**PCR**	**Baiting^a^**
Haerbin	25	16	15	13	12/11
Jiamusi	19	8	8	7	6
Qiqihaer	23	15	15	14	12/11
Mudanjiang	21	10	10	8	7
Daqing	18	10	10	8	6
Jixi	14	8	8	7	6
Yichun	10	5	5	4	3
Total	130	72	71	61	52/50

*^*a*^Two samples, one collected in Haerbin and one in Qiqihaer, were *P. sojae*-positive in the first repeat, whereas negative in the second repeat of the leaf-disc baiting experiment.*

The RPA-LFD assay along with three previously described detection methods for *P. sojae* were comparatively evaluated using the same set of 130 samples. eDNAs were used as templates in the novel RPA-LFD assay, as well as LAMP (Dai et al., 2012) and conventional PCR (Wang et al., 2006) assays. In a modified leaf-disc baiting assay (Erwin and Ribeiro, 1996; Malvick and Grunden, 2004), rhizosphere samples were dried at room temperature for 3 days. Approximately 300 g of each sample was saturated by adding distilled water and maintained under the saturated condition at room temperature for 5 days. Thirty leaf discs of soybean cultivar Hefeng 47 (2 cm in diameter) were pressed onto the surface of each saturated rhizosphere sample and incubated at room temperature in the dark for 2 to 3 days. After incubation, leaf discs were placed onto PARP selective media (containing pimarcin, ampicillin, rifampicin, and pentachloronitrobenzene) to recover isolates. Each isolate of recovered *Phytophthora* species was examined for characteristic oospores of *P. sojae*. Representative isolates were identified by sequencing the internal transcribed spacer region (Cooke et al., 2000). Each detection method was repeated once against all 130 rhizosphere samples.

## Results

### Specificity and Sensitivity of the RPA-LFD Aassay

**FIGURE 2:**
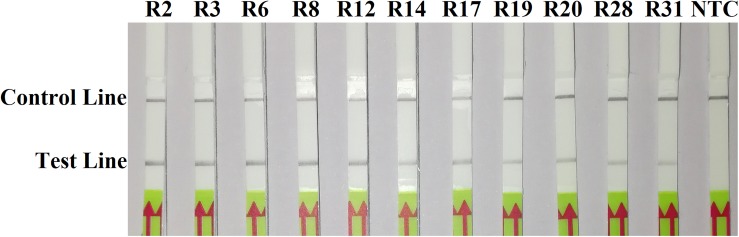
Evaluation of specificity of the novel recombinase polymerase amplification-lateral flow dipstick assay using 11 isolates belonging to different pathotypes of *Phytophthora sojae*. Nuclease-free water was used in place of DNA templates in a no-template control (NTC). Dipsticks of the first repeat are shown, as results were identical among three repeats of the experiment.

**FIGURE 3:**
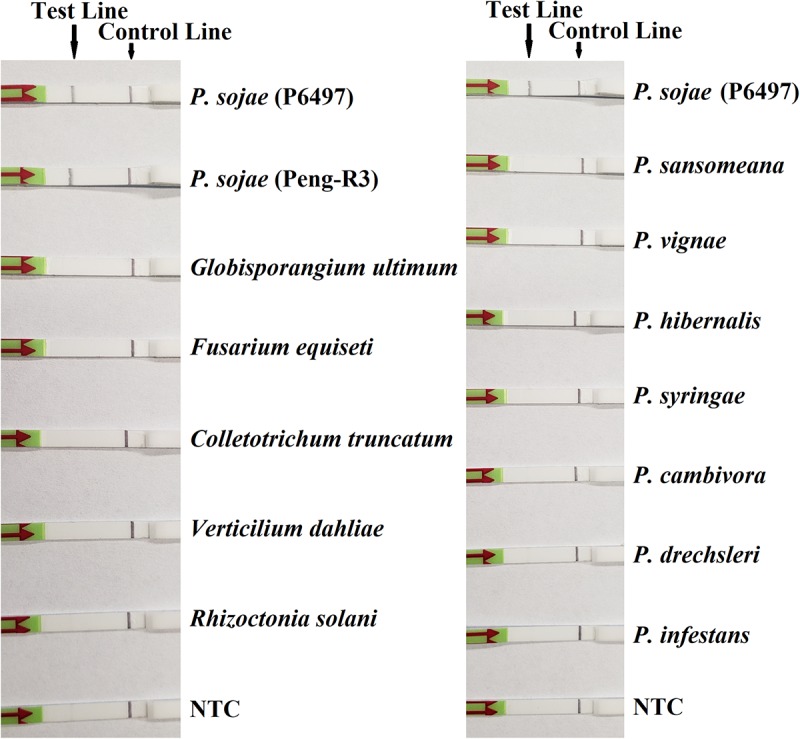
Evaluation of specificity of the novel recombinase polymerase amplification-lateral flow dipstick assay using *Phytophthora sojae* isolates and other oomycete and fungal species. Nuclease-free water was used in place of DNA templates in no-template controls (NTC). Dipsticks of selected isolates are shown. All results are listed in Table 1.

In the evaluation of specificity, identical results were observed among three repeats of the experiments. All dipsticks had a visible control line, indicating valid tests. Test lines were visible on dipsticks using gDNAs of *P. sojae* isolates. No test lines were observed on dipsticks of other species or NTC (Figures 2, 3).

**FIGURE 4:**
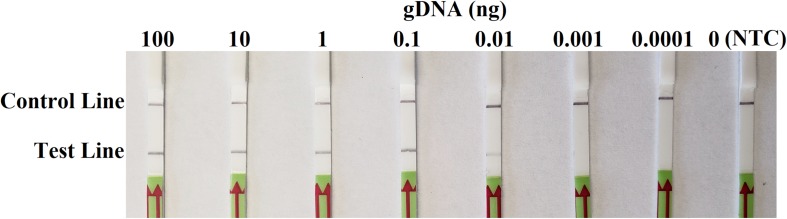
Evaluation of sensitivity of the novel recombinase polymerase amplification-lateral flow dipstick assay using 10-fold dilutions of genomic DNA (gDNA) of *Phytophthora sojae* isolate P6497 as templates. Nuclease-free water was used in place of DNA templates in a no-template control (NTC). Dipsticks of the first repeat are shown, as results were identical among three repeats of the experiment.

In sensitivity evaluation, all dipsticks had visible control lines. Test lines were visible on dipsticks correlating with 100, 10, 1, 0.1, or 0.01 ng of *P. sojae* gDNA template used per each RPA reaction. No test lines were observed on those with 0.001 or 0.0001 ng of gDNA, or NTC (Figure 4). The results at all gDNA concentrations were consistent among three repeats of the experiment.

### Detection of *P. sojae* in Artificially Inoculated Soybean Seedlings Using RPA-LFD

**FIGURE 5:**
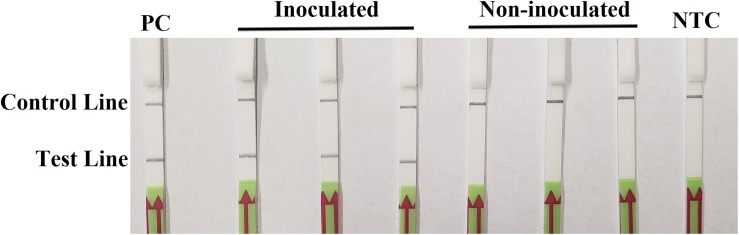
Detection of *Phytophthora sojae* in artificially inoculated soybean seedlings using the recombinase polymerase amplification-lateral flow dipstick assay. Genomic DNA (10 ng) of *P. sojae* isolate P6497 was used as the template in a positive control (PC). Nuclease-free water was used in a no-template control (NTC). Dipsticks of the first repeat are shown, as results were identical between two repeats of the experiment.

On the third day after inoculation, three inoculated seedlings had severe wilting with discoloration at wound sites. There was no discoloration on three wounded, non-inoculated hypocotyls, although a slight wilting might be observed. In the RPA-LFD assay, all dipsticks had visible control lines. Test lines were visible on three dipsticks with total DNAs extracted from inoculated hypocotyls, whereas no test lines were observed on those from three non-inoculated hypocotyls (Figure 5). Results were identical between two repeats of the experiment.

### Comparative Evaluation of Detection Assays Using Rhizosphere Samples

Detection results were identical between two repeats of the RPA-LFD, LAMP, and PCR assays. *P. sojae* was detected in 72 of 130 (55.4%) samples (Table 3) using the novel RPA-LFD assay. These 72 positive samples were collected from Haerbin (16 of 25), Jiamusi (8 of 19), Qiqihaer (15 of 23), Mudanjiang (10 of 21), Daqing (10 of 18), Jixi (8 of 14), and Yichun (5 of 10). Using the LAMP assay, 71 samples (54.6%) were detected as positive (Table 3). All 71 positive samples in the LAMP assay were also detected as positive in the RPA-LFD assay (Table 3). *P. sojae* was detected in one sample collected from Haerbin using the RPA-LFD assay, but not detectable using the LAMP assay. Using the conventional PCR assay, 61 samples (46.9%) were determined as positive. They were also positive in both RPA-LFD and LAMP assays (Table 3). The positive detection rate using PCR was lower than those of both isothermal amplification assays in each city (Table 3).

*Phytophthora sojae* was recovered from soybean leaf-disc baits deployed in 52 (40%) and 50 (38.5%) samples in two repeats of the experiment, at lower detection rates than other methods (Table 3). One sample collected from Haerbin and one from Qiqihaer were positive in the first repeat, whereas *P. sojae* was not recovered from these two samples in the second repeat of the experiment using the baiting method (Table 3).

## Discussion

Accurate and rapid detection of *P. sojae* in plants and soil is a critical step toward effective prevention and management of soybean root and crown rot and seedling damping-off. In this study, a novel method was developed to detect *P. sojae* using the RPA-LFD assay. Evaluations in the study determined this assay as specific to *P. sojae*. It was also found to be sensitive, detecting as low as 10 pg per μL of gDNA, and *P. sojae* in soil samples at a higher rate than three previously developed methods, namely LAMP (Dai et al., 2012), conventional PCR (Wang et al., 2006), and leaf-disc baiting. High sensitivity and specificity, and several other advantages make this novel RPA-LFD assay a potentially useful method in high-throughput testing under time- and resource-limited conditions.

Recombinase polymerase amplification assay in combination with LFD for the diagnosis of *P. sojae* shows a high degree of specificity. Although many previous methods were believed as *P. sojae*-specific when they were developed (Wang et al., 2006; Bienapfl et al., 2011; Haudenshield et al., 2017), their accuracy has been challenged by newly emerging pathogens (Rojas et al., 2017; Xiong et al., 2019), such as *P. sansomeana* (Hansen et al., 2009), another species pathogenic to soybeans, and phylogenetic sister taxa of *P. sojae*, such as *P. melonis* and *P. vignae*. Rojas et al. (2017) reported that an RPA assay targeting the mitochondrial *atp9*–*nad9* region was specific to the genus *Phytophthora* and several species including *P. sojae*. This high specificity has also been found in the novel RPA-LFD assay targeting *Ypt1* gene in this study. As demonstrated in the specificity evaluation, this novel RPA-LFD assay detected DNAs of *P. sojae*, while had no positive reactions to those of 24 other *Phytophthora* species, including *P. sansomeana*, *P. melonis*, and *P. vignae* (Table 1).

Sensitivity of RPA-LFD assay reported here is adequate if not higher than most previously developed methods. In the sensitivity evaluation using gDNA, the detection lower limit for this RPA-LFD assay was 0.01 ng (10 pg) in a 50 μL RPA reaction (Figure 4). It was at least 100 and 10 times more sensitive than a conventional PCR assay (Wang et al., 2006) and *Ypt1*-based LAMP assay (Dai T.T. et al., 2015), respectively, and equally sensitive as an *A3aPro*-specific LAMP assay (Dai et al., 2012). In the comparative evaluation using field soil samples, the RPA-LFD assay resulted in the highest detection rate of *P. sojae* among four evaluated methods (Table 3). The only higher sensitivity reported so far was 100 fg in a PCR-based method using a set of four SCAR primers (Xiong et al., 2019). However, RPA has the advantage in using fewer primers and special equipment, as well as its significantly shorter amplification time.

Several advantages make this RPA-LFD assay useful under time- and resource-limited conditions. First, RPA reaction does not require specialized equipment such as LAMP devices, PCR thermal cyclers, or electrophoresis systems. Second, RPA reactions could be performed within a wider temperature range between 25 and 45°C (James and Macdonald, 2015; Daher et al., 2016). In contrast, PCR-based methods require stringent control of various temperatures, while LAMP assays require a consistently high temperature for amplification, approximately 64°C. Third, the RPA-LFD assay is a time-saving diagnostic tool. This two-step assay only requires 20 min for RPA and less than 5 min for LFD detection. The reaction durations usually double for LAMP assays and are at least 90 min for PCR. Fourth, the RPA-LFD assay does not require a fluorometer to monitor the fluorescent signal. RPA results can be directly visualized on the dipsticks, making this method much simpler to operate than any other methods. Due to the rapid disease development and field-to-field spread of *P. sojae* (Erwin and Ribeiro, 1996), simplicity and time-saving are important merits of diagnostic tools, especially when disease prevent and pathogen eradication are urgent and a large quantity of samples are required to be processed. Fifth, RPA assays are more resistant to inhibitors such as host DNA as compared to other isothermal detection methods, such as LAMP, although false negative results can also occur (Rosser et al., 2015; Moore and Jaykus, 2017; Ahmed et al., 2018). In this study, total DNAs containing both pathogen and host gDNAs were extracted from *P. sojae*-inoculated soybean hypocotyls, while no false negative result was yielded (Figure 5). This finding indicated that inhibitory effects of soybean gDNA was unlikely involved in the present RPA assay.

A pipeline framework of developing a novel RPA-LFD assay for a specific *Phytophthora* species has been demonstrated in this study, including designing specific RPA primers, optimizing reaction conditions of RPA and LFD visualization, and evaluating the assay’s sensitivity and specificity. The unique sequence of the *Ypt1* gene of *P. sojae* was targeted here, while other genetic markers could be utilized for developing isothermal amplification assays for *P. sojae* (Dai et al., 2012) and other plant pathogens. With the increasing availability of genome sequences, identification of species-specific markers has become easier and more affordable. For example, a comparative genomics approach has been applied for designing LAMP primers specific to *Phytophthora cinnamomi* (Dai et al., 2019). A similar approach has been used for developing a *Pectobacterium* species-specific RPA-LFD assay (Ahmed et al., 2018). It is not unexpected that additional RPA assays using a diverse of genetic markers will be developed for detecting an array of important *Phytophthora* species in the future.

## Conclusion

A novel RPA-LFD assay was developed for the accurate, simple and rapid detection of *P. sojae*. The specific primers combination was determined by targeting the *Ypt1* gene. The RPA-LFD assay could perform at the temperature range of 25–45°C within 25 min. This assay has several notable advantages. Only a primer pair plus a probe are required to detect trace amounts of DNA. Meanwhile, the amplicons could generate visible lines on LFD, while no gel electrophoresis is required. Additionally, sensitivity evaluation revealed that RPA-LFD assay could detect as low as 10 pg gDNA of *P. sojae*. Furthermore, the RPA-LFD assay successfully detected *P. sojae* in inoculated plant tissues and infested soil samples at higher rates than LAMP, PCR, and leaf-disc baiting methods. Based on the above findings, this RPA-LFD assay has great potential to be adapted as a routine test for detecting *P. sojae*, especially under time- and resource-limited conditions.

## Data Availability

The raw data supporting the conclusions of this manuscript will be made available by the authors, without undue reservation, to any qualified researcher.

## Author Contributions

TD, XY, XZ, and DS conceived and designed the experiments, contributed the reagents, materials and analysis tools, and wrote the manuscript. TH, BJ and YX performed the experiments and analyzed the data.

## Conflict of Interest Statement

The authors declare that the research was conducted in the absence of any commercial or financial relationships that could be construed as a potential conflict of interest.

## Footnotes

1 http://www.aqsiq.gov.cn/; June 22, 2016
